# The impact of Adult Comorbidity Evaluation-27 on the clinical outcome of elderly nasopharyngeal carcinoma patients treated with chemoradiotherapy or radiotherapy: a matched cohort analysis

**DOI:** 10.7150/jca.35311

**Published:** 2019-09-07

**Authors:** Yue-Feng Wen, Xue-Song Sun, Li Yuan, Li-Si Zeng, Shan-Shan Guo, Li-Ting Liu, Chao Lin, Hao-Jun Xie, Sai-Lan Liu, Xiao-Yun Li, Yi-Bin Zhang, Wen-Jin Huang, Hai-Hua Peng, Zhi-Wei Liao, Xian-Lu Song, Qing-Nan Tang, Yu-Jing Liang, Jin-Jie Yan, Jin-Hao Yang, Zhen-Chong Yang, Qiu-Yan Chen, Xiao-Dan Lin, Lin-Quan Tang, Hai-Qiang Mai

**Affiliations:** 1 State Key Laboratory of Oncology in South China, Collaborative Innovation Center for Cancer Medicine, Guangdong Key Laboratory of Nasopharyngeal Carcinoma Diagnosis and Therapy, Sun Yat-sen University Cancer Center, Guangzhou 510060, P. R. China; 2 Department of Nasopharyngeal Carcinoma, Sun Yat-Sen University Cancer Center, 651 Dongfeng Road East, Guangzhou 510060, P. R. China; 3 Department of Radiotherapy, Affiliated Cancer Hospital & Institute of Guangzhou Medical University, 78 Hengzhigang Road, Guangzhou 510095, P. R. China; 4 Cancer Research Institute, Affiliated Cancer Hospital & Institute of Guangzhou Medical University, 78 Hengzhigang Road, Guangzhou 510095, P. R.China

**Keywords:** Nasopharyngeal carcinoma, Elderly, Chemoradiotherapy, Intensity-modulated radiotherapy, Adult Comorbidity Evaluation-27

## Abstract

**Objectives**: To evaluate the prognostic significance of Adult Comorbidity Evaluation-27 (ACE-27) for elderly patients (age ≥70 years) with locoregionally advanced nasopharyngeal carcinoma (NPC) treated with Intensity-Modulated Radiotherapy (IMRT), with or without chemotherapy.

**Methods**: 206 elderly patients with locoregionally advanced NPC treated from December 2006 to December 2016 were involved into analysis as the training cohort. Besides, a separate cohort of 72 patients from the same cancer center collected between January 2003 and October 2006 served as the validation cohort. By using propensity score matching (PSM), we created a balanced cohort by matching patients who received chemoradiotherapy with patients who received IMRT alone. Treatment toxicities were calculated between CRT and RT groups using the χ^2^ test. The primary endpoint was cancer-specific survival (CSS). Multivariate analysis was performed to assess the relative risk for each factor by using a Cox's proportional hazards regression model.

**Results**: The median follow-up was 39.0 months (range = 3-137 months). In the PSM cohort, patients in the CRT group achieved comparable survival compared with patients in the RT group. The 3-year CSS rate was 64.3% and 65.2%, respectively (P =0.764). In multivariate analysis, the addition of chemotherapy to IMRT was not an independent prognostic factor for CSS, whereas a high ACE-27 score was an independent risk factor. In subgroup analysis with ACE-27 score ≥ 2, the 3-year CSS rate was worse in patients from the CRT group (63.5% vs. 46.3%, P = 0.041).

**Conclusions**: CRT is comparable to IMRT alone for elderly patients with locoregionally advanced NPC. The ACE-27 tool may help to identify high-risk subgroup for poor disease outcome and tailor individualized treatment.

## Background

Nasopharyngeal carcinoma (NPC) is endemic in Southern China and Southeast Asia 1. It has been well established by a variety of prospective randomised trials and meta-analyses that concurrent chemoradiotherapy (CCRT) with or without adjuvant chemotherapy is superior to radiotherapy (RT) alone in treating locoregionally advanced NPC 2-6. Consequently, CCRT with or without adjuvant chemotherapy has been recommended as the standard treatment regimen for locoregionally advanced NPC. However, since elderly patients are often accompanied with poorer performance status, multiple comorbidities and decreasing organ function, they are commonly excluded from clinical trials. Thus, the treatment guidelines are principally tailored for younger NPC patients.

A population-based analysis demonstrated older age at diagnosis was associated with a higher risk of NPC-related mortality 7. Previous studies also reported about the higher rate of severe chemotherapy-related toxicities and inferior survival observed in patients with comorbidities 8, 9. More severe comorbidities were considered a predictor of poorer survival in various cancers, such as laryngeal squamous cell carcinoma 10, non-small-cell lung cancer (NSCLC) 11, breast cancer 12, 13 , and colorectal cancer 14.

The Adult Comorbidity Evaluation-27 (ACE-27) tool is a chart-based assessment method specific for the evaluation of comorbidity in patients with cancer 9, 15. It was found by Sze et al. that elderly NPC patients, who had an ACE-27 score ≥2 after radical radiotherapy were associated with worse survival outcomes than their counterparts with an ACE-27 score <2 16. Comorbidity may exert an adverse effect on survival outcomes of cancer patients by acting as a competing cause of death or by impairing use, tolerability, or effectiveness of treatments such as chemotherapy 8. Therefore, a reliable assessment of comorbidity is required to distinctly estimate the potential benefits and risks of chemotherapy.

Since intensity-modulated radiation therapy (IMRT) has shown excellent locoregional control in NPC, the survival benefit brought by chemotherapy has been impaired in comparison. Distant metastasis remains the most difficult treatment challenge for elderly patients with NPC 17. Yang et al. demonstrated that CCRT was favorable for NPC patients aged ≥60 years with high EBV DNA levels but not for subgroups with low EBV DNA levels 18. In a subgroup analysis of 57 cases of elderly patients (age ≥70 years) with stage I- IV NPC and an ACE-27 score ≥2, Jin et al. showed that CRT reduced the overall survival (OS) rate for this subgroup with a borderline significance, compared with IMRT treatment on its own 19. However, this study was limited by small sample size and its retrospective nature, thus a matched cohort study with a larger sample size would be better suited to confirm the efficacy of chemotherapy on elderly patients. Furthermore, such a study would better elucidate which subgroup benefit most from chemotherapy. It is not clear whether or not chemotherapy contribute to expanding the life span of elderly NPC patients in the IMRT era. Therefore, we performed a two-centre matched retrospective analysis of a relatively large cohort to identify the prognostic value of ACE-27 for risk stratification, and also investigated the efficacy of chemotherapy on stage III-IV NPC patients older than 70 years.

## Methods

### Patients

**Figure 1:**
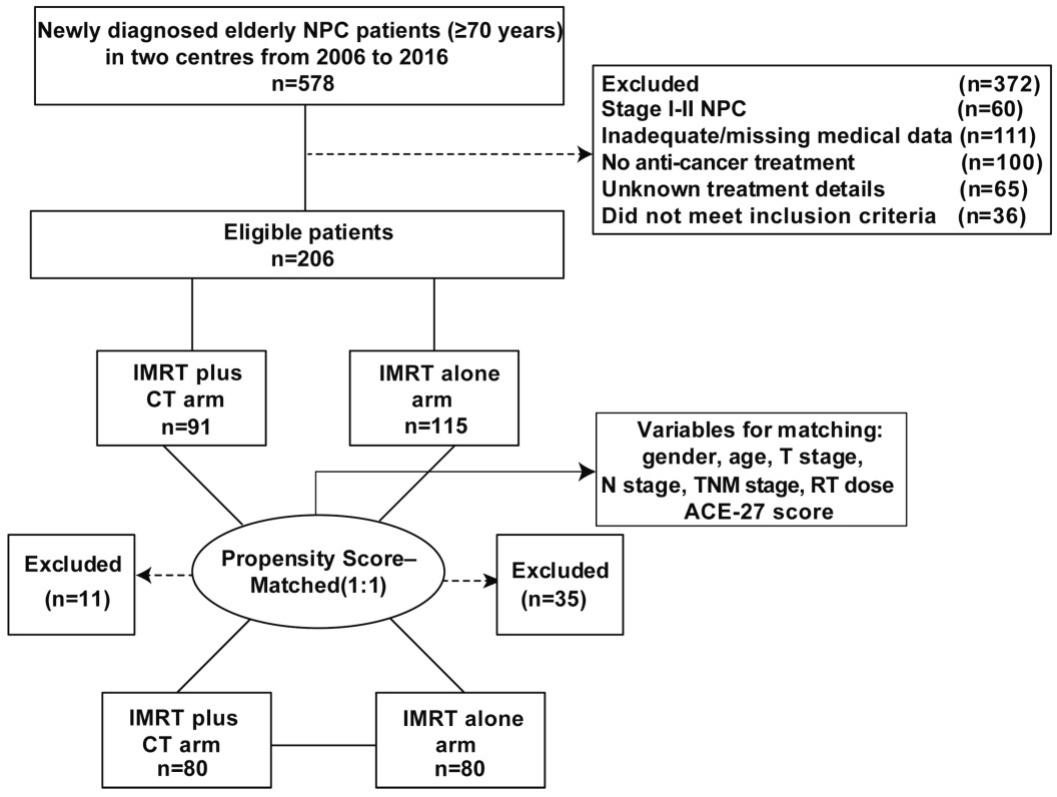
Flow chart of patients inclusion.

A total of 578 elderly patients (age ≥70 years) with primary NPC were consecutively recruited from December 2006 to December 2016 at the Sun Yat-Sen University Cancer Centre and the Cancer Centre of Guangzhou Medical University, China. Among them, 206 NPC patients were included in this study. The inclusion criteria were as follows: newly diagnosed NPC without metastasis; age ≥70 years; stage III-IVb disease (the 7^th^ American Joint Committee on Cancer/Union for International Cancer Control (AJCC/UICC) staging system); completed radical IMRT at the end; chemotherapy regimens included cisplatin-based concurrent chemotherapy with or without sequential chemotherapy (e.g., neoadjuvant and/or adjuvant chemotherapy); no previous history of previous chemotherapy or RT; completed follow-up after treatment. Additionally, we recruited 72 elderly patients with locoregionally advanced NPC at the same institute between January 2003 and October 2006, which was served as the validation cohort. Figure 1 showed the patient flowchart. This study was approved by the clinical research ethics committee of the Sun Yat-Sen University Cancer Centre and the Cancer Centre of Guangzhou Medical University. All participants provided written informed consent.

### Pretreatment assessment and treatment

All patients received a physical examination, complete blood count, liver and renal biochemistries, fiber-optic endoscopy of the nasopharynx, chest radiograph, abdominal ultrasonography, magnetic resonance imaging (MRI) or computed tomography (CT) of the nasopharynx and neck region, and a bone scan by emission computed tomography (ECT). All patients were re-staged based on the 7th AJCC/UICC staging system. We performed the ACE-27 assessments according to our previous study 20 and followed the principles of the Comorbidity Coding Book (Additional file 1). ACE-27 score at presentation was retrospectively determined by review of medical records. The Common Terminology Criteria for Adverse Events (CTCAE) version 4.0 was used to grade treatment-related acute toxicities. Acute and late radiation related complications were graded according to the Radiation Therapy Oncology Group/European Organization for Research and Treatment of Cancer (RTOG/EORTC) Late Radiation Morbidity Scoring Schema 23. All patients were treated in accordance with principles of treatment for NPC patients at SYSUCC. More detailed information on treatment is available in Supplementary Materials (available online).

### Follow-up

The primary end-point of this study was cancer-specific survival (CSS), which was defined as the duration from the date of treatment commencement to the date of death as a result of NPC or censored at the date of last follow-up. The secondary end-points included overall survival (OS), progression-free survival (PFS), locoregional relapse-free survival (LRRFS), and distant metastasis-free survival (DMFS). OS was defined as the time from the start date of treatment to the date of death from any cause or censored at the date of last follow-up. PFS was defined as the time from the start date of treatment to the date of relapse at any site, death from any cause, or censored at the date of last follow-up. LRRFS and DMFS were defined as the time from the start date of treatment to the date of first locoregional or distant relapse, or censored at the date of last follow-up. Follow-up duration was calculated from the start date of treatment to the date of the observed endpoints or to the date of last follow-up.

### Statistical methods

The Pearson χ^2^ test or Fisher's exact test was used for non-parametric variables. Propensity score matching (PSM) was performed to control for pretreatment imbalances on observed variables 24, 25. Patients who received CRT were matched to patients treated with IMRT alone by using a 1:1 matching approach according to age <75 years and ≥75 years), gender (male and female), T stage (stage T1-2 and stage T3-4), N stage (stage N0-1 and stage N2-3), TNM stage (stage III and IVa-b), RT dose for nasopharynx (<70Gy and ≥70Gy), RT dose for lymph node (<60Gy and ≥60Gy), and ACE-27 scores (<2 and ≥2). Survival probabilities were calculated using the Kaplan-Meier method. The log-rank test was used to analyze for significant differences between the survival curves. Multivariate analysis was performed using a Cox's proportional hazards regression model with a forward stepwise procedure (the entry and removal probabilities were 0.05 and 0.10, respectively). Analyses were performed using the statistical software package SPSS, version 23.0 (SPSS, Chicago, IL, USA). A two-sided P-value less than 0.05 was considered statistically significant.

## Results

### Patient characteristics

From December 2006 to December 2016, we retrospectively included 206 stage III-IV NPC patients over 70 years old in the training cohort. Clinical characteristics of the whole cohort were listed in Table 1. There were 91 cases in the CRT group and 115 cases in the RT group. The male-to-female ratio was 3.6:1 and the median age of all patients was 73 years old. There was a significantly higher proportion of patients aged over 75 years and TNM stage III in patients without chemotherapy (P < 0.001 and P = 0.002, respectively). Thereupon, we matched the two groups for all potential prognostic factors at a ratio of 1:1 to reduce potential bias. Finally, a well-balanced cohort was created with 160 patients (all P > 0.05) (Table 1). From January 2003 to October 2006, seventy-two elderly patients with locoregionally advanced NPC at the same institute were served as the validation cohort. The validation cohort was also created by PSM method and the characteristics of patients were shown in Table S1.

### Survival analysis

**Figure 2:**
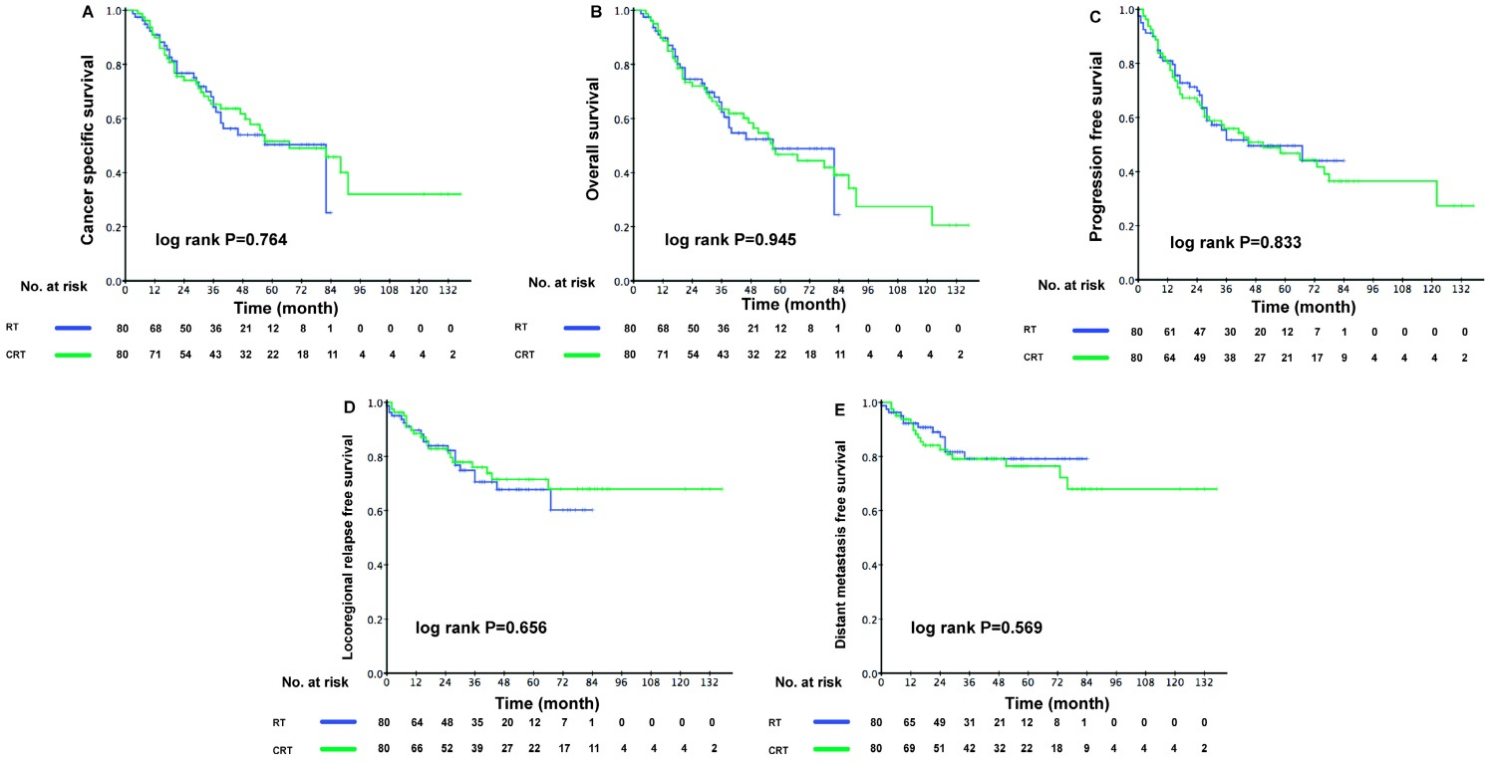
Kaplan-Meier survival curves between the RT and CRT groups in elderly patients. Shown are results for (A) cancer-specific survival, (B) overall survival, (C) progression-free survival, (D) locoregional relapse-free survival, (E) distant metastasis free survival. P values were calculated using the unadjusted log-rank test.

Median follow-up was 39.0 months (range = 3-137 months) and 96 patients died during follow-up. The 1-year, 3-year, and 5-year OS were 90.7%, 69.4%, and 51.4%, respectively. However, when patients were grouped by treatment method, the addition of chemotherapy to IMRT failed to boost the survival outcomes of patients from the PSM cohort. The 3-year CSS was 64.3% (95% confidence interval [CI], 52.5-76.1%) in the RT group and 65.2% (95% CI, 54.2-76.2%) in the CRT group (P = 0.764). No significant difference was detected in other clinical endpoints assessed. The Kaplan-Meier curves were shown in Figure 2. These results were further confirmed in the validation cohort (Supplementary Figure S1). Furthermore, we conducted multivariate analysis for all 206 eligible patients. After adjusting for all potential prognostic variables, we found that the application of chemotherapy was not a significantly independent prognostic factor of CSS (CRT vs. RT: HR, 1.221; 95% CI, 0.752-1.985; P = 0.419). However, patients with stage IV disease exhibited a higher risk of cancer specific death (stage IV vs. stage III: HR, 2.240; 95% CI, 1.418-3.537; P = 0.001). Moreover, a high ACE-27 score was an independent risk determinant for CSS (ACE-27 score ≥ 2 vs. ACE-27 score < 2: HR, 2.359; 95% CI, 1.524-3.652; P < 0.001) (Table 2).

### Toxicities

The effect of toxicity in each group was shown in Table 3. The rates of G2-G3 hematological toxicities (leukocytopaenia, neutropaenia, anaemia, and thrombocytopaenia) were higher in the CRT group compared with the RT group. Moreover, a significantly higher frequency of G2-3 nausea and vomiting (27.5% vs. 2.6%, P < 0.001) was noted in patients receiving chemotherapy. The incidences of skin reactions and mucositis were similar in both groups. However, the CRT group was found to develop hepatotoxicity with higher chances. No noticeable distinction between the treatment groups were observed in terms of late toxicities including deafness, xerostomia, neck fibrosis and trismus.

### Subgroup analysis

**Figure 3:**
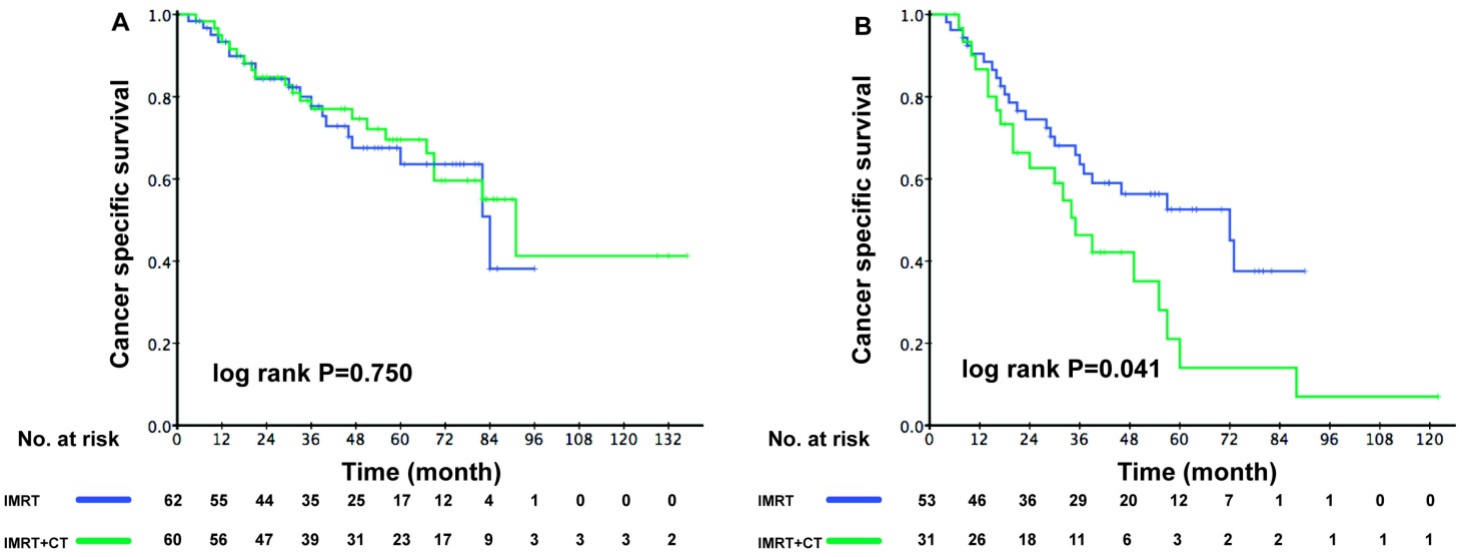
Kaplan-Meier survival curves between the RT and CRT groups for cancer -specific survival in elderly patients. (A) Low-risk patients (ACE-27 score 0-1), (B) High-risk patients (ACE-27 score 2-3).

The ACE-27 score was an independent prognostic factor for elderly patients with locoregionally advanced NPC, therefore we divided the patients into low-risk and high-risk group accordingly. In the low-risk group, the 3-year CSS rate was 77.7% in the RT group and 77.7% in the CRT group (P = 0.750). However, the 3-year CSS rate was poorer in high-risk patients from the CRT group (63.5% vs. 46.3%, P = 0.041) (Figure 3A-B).

## Discussion

To the best of our knowledge, this is the first matched cohort study from two centers to explore whether CRT is superior to RT in locoregionally advanced NPC patients aged ≥70 years based on different risk stratification of ACE-27 scores in the IMRT era. Since the elderly generally present with one or more comorbidities and are more inclined to die from the comorbidities rather than the tumor itself, CSS could be a more accurate and specific prognostic indicator than OS without influence from other factors. In the present study, we could not demonstrate that the addition of chemotherapy to IMRT could improve survival in locoregionally advanced NPC patients aged ≥70 years. Nonetheless, ACE-27 and TNM stage played critical roles in predicting survival of elderly patients with locoregionally advanced NPC. An ACE-27 score ≥ 2 revealed poorer CSS. Furthermore, in the subgroup analysis of patients with ACE-27 score ≥2, CRT was significantly associated with unsatisfactory survival compared with IMRT alone.

A previous retrospective analysis which enrolled 126 patients older than 70 years with stage I-IV NPC showed that there was no superiority in CRT compared with IMRT alone. Additionally, a subgroup analysis of patients with ACE-27 score ≥2 was performed to further confirm the OS benefit from CRT in this study but unfortunately, due to the small sample size of 57 patients, only borderline significance was demonstrated 19. Our findings were consistent with the aforementioned evidences, with the exception that after PSM analysis, the CRT group had a worse survival outcome than the RT group because of a higher rate of grade 3-4 acute toxicities observed in the patients with ACE-27 ≥2. Yang et al. illustrated that CCRT was beneficial for stage II-IVB NPC patients aged ≥60 years 18. However, only 23.7% of patients had stage II NPC in the aforementioned study, whereas our study was conducted within a stage-specific cohort and only enrolled elderly patients aged ≥70 years with stage III-IVB NPC. Such a methodological difference may account for the observed inconsistency in treatment outcomes between the two studies.

The effect of ACE-27 tool for prognostic risk assessment and treatment stratification has been investigated in various cancers 9, 11-13, 16, 26-29. A retrospective study from England failed to reveal the significant effect of ACE-27 on the prognosis of 59 NPC patients but this study was underpowered due to its small sample size 28. Sze et al. identified ACE-27 as the only predictive variable for mortality at 90 days and the most important prognostic factor for OS in NPC patients aged >70 years. However, no more than 30% patients in this study were treated with IMRT 16. Giacalone et al. suggested that the recommendation of treatment on the basis of reduced Prostate-Specific Antigen (PSA) failure derived from early results of randomized controlled trials (RCTs), and while it was unlikely to prolong survival in men with moderate-to-severe comorbidity, it may extend survival in men with no or minimal comorbidity 29. Our study confirmed that the ACE-27 score is an independent prognostic factor for the survival of elderly patients with locoregionally advanced NPC after IMRT. Therefore, the utility of ACE-27 should be taken into account with respect to patient selection and treatment strategy.

In the present study, we performed a subgroup analysis based on different risk stratification of the ACE-27 scores. We revealed a negative association between CRT and CSS-/OS-related survival in elderly patients with an ACE-27 score ≥2 compared with those received IMRT alone. In the meantime, elderly patients with an ACE-27 score <2 showed similar survival benefits between the two treatment regimens. The following evidence may provide further support to these data. Patients with comorbidities may be more prone to suffer from severe treatment-related toxicities, dose delays or reductions, therefore it is possible they do not survive long enough to derive expected benefits from chemotherapy 8. Given more severe acute toxicity, poorer tolerance, and worse survival outcomes, chemoradiotherapy should only be considered in elderly patients aged ≥70 years with locoregionally advanced NPC, especially in those patients with severe comorbidities. Therefore, it is of necessity to perform a comprehensive assessment of comorbidity in elderly patients afflicted by NPC before treatment. Relatively safe and effective therapeutic strategies such as vascular endothelial growth factor (VEGF) inhibitor, Epstein-Barr virus (EBV)-specific vaccines, along with novel immunotherapies targeting immune checkpoints may offer promising alternatives for elderly patients with locoregionally advanced NPC.

There are some limitations to our study. Firstly, the median follow-up duration was 39.0 months, and a longer follow-up duration is in need to observe the survival status beyond this. Secondly, due to the retrospective nature of the study, potential patient selection bias is unavoidable. What's more, EBV DNA concentration is one of the most important prognostic factors for the survival of NPC. However, patients with EBV DNA copy data only account for a small proportion of those in this study (data not shown). Therefore, further prospective randomized clinical trials should be conducted to confirm the effect of chemotherapy on elderly patients aged ≥70 years treated with IMRT.

## Conclusion

An ACE-27 score ≥2 was significantly associated with poor CSS. Chemotherapy added to IMRT could not improve survival for locoregionally advanced NPC patients aged ≥70 years. In the subgroup analysis of patients with an ACE-27 score ≥2, the CRT group had poorer survival compared with the RT group.

## Supplementary Material

Supplementary methods, figure, and table.

## Figures and Tables

**Table 1:** Clinical characteristics

	Primary cohort (n=206)			PSM cohort (n=160)		
Characteristics	CRT	RT	P-value	CRT	RT	P-value
**Total**	115	91		80	80	
**Age, y**						
<75	63(54.8%)	77(84.6%)	<0.001	55(68.8%)	66(82.5%))	0.065
≥75	52(45.2%)	14(15.4%)		25(31.3%)	14(17.5%)	
**Gender**						
Female	27(23.5%)	18(19.8%)	0.611	18(22.5%)	17(21.3%)	1.000
Male	88(76.5%)	73(80.2%)		62(77.5%)	63(78.8%)	
**T stage#**						
T1-2	9(7.8%)	11(12.1%)	0.348	8(10.0%)	11(13.8%)	0.626
T3-4	106(92.2%)	80(87.9%)		72(90.0%)	69(86.3%)	
**N stage#**						
N0-1	57(49.6%)	43(47.3%)	0.780	39(48.8%)	32(40.0%)	0.340
N2-3	58(50.4%)	48(52.7%)		41(51.2%)	48(60.0%)	
**TNM stage#**						
III	77(67.0%)	51(45.1%)	0.002	42(52.5%)	41(51.2%)	1.000
IV	38(33.0%)	50(54.9%)		38(47.5%)	39(48.8%)	
**RT dose (NP)**						
<70 Gy	4(3.5%)	1(1.1%)	0.386*	2(2.5%)	1(1.3%)	1.000*
≥70 Gy	111(96.5%)	90(98.9%)		78(97.5%)	79(98.8%)	
**RT dose (LN)**						
<60 Gy	38(33.0%)	25(27.5%)	0.447	25(31.3%)	25(31.3%)	1.000
≥60 Gy	77(67.0%)	66(72.5%)		55(68.8%)	55(68.8%)	
**ACE-27 score**						
0-1	62(53.9%)	60(65.9%)	0.088	49(61.3%)	50(62.5%)	1.000
2-3	53(46.1%)	31(34.1%)		31(38.8%)	30(37.5%)	

Abbreviations: PSM = propensity score matching; IMRT = intensity modulated radiotherapy; CRT = chemoradiotherapy; RT = radiotherapy; NP = nasopharynx; LN = lymph node; ACE-27 = Adult Comorbidity Evaluation-27 # According to the 7th edition of UICC/AJCC staging system P-value was calculated using the Pearson χ^2^ test or Fisher's exact test (*)

**Table 2:** Multivariable analysis of prognostic factors for CSS, OS, PFS, LRRFS, and DMFS (n = 206)

Characteristics	HR	*95%CI*	*P value*
**Cancer specific survival**			
Age	1.726	1.071-2.781	0.025
Gender	2.261	1.205-4.242	0.011
T stage	1.482	0.643-3.417	0.356
N stage	1.388	0.871-2.213	0.168
TNM stage	2.240	1.418-3.537	0.001
RT dose (NP)	0.830	0.185-3.720	0.808
RT dose (LN)	0.944	0.591-1.506	0.808
ACE-27 score	2.359	1.524-3.652	<0.001
Treatment method	1.221	0.752-1.985	0.419
**Overall survival**			
Age	1.681	1.072-2.636	0.024
Gender	2.399	1.330-4.327	0.004
T stage	1.555	0.712-3.400	0.268
N stage	1.613	1.030-2.525	0.037
TNM stage	2.152	1.398-3.312	<0.001
RT dose (NP)	0.331	0.117-0.934	0.037
RT dose (LN)	1.073	0.679-1.695	0.764
ACE-27 score	2.350	1.556-3.550	<0.001
Treatment method	1.265	0.799-2.001	0.315
**Progression free survival**			
Age	1.486	0.961-2.297	0.075
Gender	1.960	1.139-3.372	0.015
T stage	1.380	0.663-2.872	0.389
N stage	1.651	1.063-2.564	0.026
TNM stage	1.714	1.136-2.586	0.010
RT dose (NP)	0.496	0.179-1.377	0.178
RT dose (LN)	1.030	0.662-1.601	0.896
ACE-27 score	2.356	1.576-3.524	<0.001
Treatment method	1.264	0.813-1.964	0.298
**Loco-regional relapse-free survival**			
Age	1.859	1.051-3.290	0.033
Gender	1.193	0.611-2.331	0.605
T stage	0.872	0.344-2.210	0.773
N stage	1.050	0.573-1.992	0.875
TNM stage	2.072	1.185-3.625	0.011
RT dose (NP)	0.746	0.157-3.545	0.712
RT dose (LN)	1.002	0.548-1.834	0.994
ACE-27 score	2.715	1.562-4.717	<0.001
Treatment method	1.159	0.641-2.096	0.626
**Distant metastasis-free survival**			
Age	0.911	0.400-2.074	0.824
Gender	2.903	1.006-8.378	0.049
T stage	2.760	0.630-2.100	0.178
N stage	2.462	1.197-5.064	0.014
TNM stage	1.264	0.627-2.547	0.513
RT dose (NP)	--	--	--
RT dose (LN)	0.664	0.333-1.322	0.244
ACE-27 score	1.707	0.868-3.357	0.121
Treatment method	1.386	0.654-2.938	0.394

Abbreviations: HR = hazard ratio; CI = confidence interval; NP = nasopharynx; LN = lymph node; CSS = cancer specific survival; OS = overall survival; PFS = progression free survival; LRRFS = loco-regional relapse-free survival; DMFS = distant metastasis-free survival; ACE-27 = Adult Comorbidity Evaluation-27. A Cox proportional hazard model was used to perform multivariate analyses. All variables were transformed into categorical variables. HRs were calculated for Age (years) (≥75 vs. <75); Gender (Male vs. Female); T stage (T3-4 vs. T1-2); N stage (N2-3 vs. N0-1); TNM stage (VI vs. III); RT dose (NP) (≥70 Gy vs. <70 Gy); RT dose (LN) (≥60 Gy vs. <60 Gy); ACE-27 score (2-3 vs. 0-1) and Treatment method (CRT vs. RT alone) -- HR could not be calculated as no incident occurred in the RT dose (NP) <70 Gy subgroup

**Table 3:** Acute and chronic toxicities

Toxic effect	RT	CRT	P value
**Leukocytopaenia**			
G0	68(59.1%)	15(16.5%)	<0.001*
G1	37(32.2%)	31(34.1%)	
G2	10(8.7%)	37(40.7%)	
G3	0(0.0%)	8(8.8%)	
**Neutropaenia**			
G0	100(87.0%)	52(57.1%)	<0.001*
G1	12(10.4%)	19(20.9%)	
G2	3(2.6%)	18(19.8%)	
G3	0(0.0%)	2(2.2%)	
**Anaemia**			
G0	104(90.4%)	57(62.6%)	<0.001
G1	10(8.7%)	25(27.5%)	
G2	1(0.9%)	9(9.9%)	
**Thrombocytopaenia**			
G0	115(100.0%)	82(90.1%)	0.001*
G1	0(0.0%)	7(7.7%)	
G2	0(0.0%)	1(1.1%)	
G3	0(0.0%)	1(1.1%)	
**Nausea and vomiting**			
G0	107(93.0%)	42(46.2%)	<0.001
G1	5(4.3%)	24(26.4%)	
G2	3(2.6%)	16(17.6%)	
G3	0(0.0%)	9(9.9%)	
**Skin reaction**			
G0	1(0.9%)	2(2.2%)	0.422*
G1	21(18.3%)	11(12.1%)	
G2	83(72.2%)	66(75.5%)	
G3	10(8.7%)	12(13.2%)	
**Mucositis**			
G0	1(0.9%)	1(1.1%)	0.085*
G1	38(33.0%)	18(19.8%)	
G2	39(33.9%)	30(33.0%)	
G3	37(32.2%)	41(45.1%)	
G4	0(0.0%)	1(1.1%)	
**Hepatoxicity**			
G0	102(88.7%)	69(75.8%)	0.013
G1	9(7.8%)	20(22.0%)	
G2	3(2.6%)	2(2.2%)	
G3	1(0.9%)	0(0.0%)	
**Nephrotoxicity**			
G0	114(99.1%)	87(95.6%)	0.172*
G1	1(0.9%)	4(4.4%)	
**Deafness**			
G0-1	85(73.9%)	68(73.7%)	0.349*
G2	23(20.0%)	13(14.3%)	
G3	7(6.1%)	9(9.9%)	
G4	0(0.0%)	1(1.1%)	
**Xerostomia**			
G0	53(46.1%)	52(57.1%)	0.111
G1	58(50.4%)	33(36.3%)	
G2	4(3.5%)	6(6.6%)	
**Neck fibrosis**			
G0	83(72.2%)	69(75.8%)	0.504*
G1	26(22.6%)	16(17.6%)	
G2	5(4.3%)	3(3.3%)	
G3	1(0.9%)	3(3.3%)	
**Trismus**			
G0	99(86.1%)	80(87.9%)	0.241*
G1	8(7.0%)	8(8.8%)	
G2	7(6.1%)	1(1.1%)	
G3	1(0.9%)	2(2.2%)	

P-value was calculated using the Pearson χ^2^ test or Fisher's exact test (*) Abbreviations: CRT = chemoradiotherapy; RT = radiotherapy

## References

[1] Wee JT, Ha TC, Loong SL, Qian CN (2010). Is nasopharyngeal cancer really a "Cantonese cancer". Chin J Cancer.

[2] Blanchard P, Lee A, Marguet S, Leclercq J, Ng WT, Ma J, Chan AT, Huang PY, Benhamou E, Zhu G, Chua DT, Chen Y, Mai HQ, Kwong DL, Cheah SL, Moon J, Tung Y, Chi KH, Fountzilas G, Zhang L, Hui EP, Lu TX, Bourhis J, Pignon JP (2015). Chemotherapy and radiotherapy in nasopharyngeal carcinoma: an update of the MAC-NPC meta-analysis. Lancet Oncol.

[3] Al-Sarraf M, LeBlanc M, Giri PG, Fu KK, Cooper J, Vuong T, Forastiere AA, Adams G, Sakr WA, Schuller DE, Ensley JF (1998). Chemoradiotherapy versus radiotherapy in patients with advanced nasopharyngeal cancer: phase III randomized Intergroup study 0099. J Clin Oncol.

[4] Wee J, Tan EH, Tai BC, Wong HB, Leong SS, Tan T, Chua ET, Yang E, Lee KM, Fong KW, Tan HS, Lee KS, Loong S, Sethi V, Chua EJ, Machin D (2005). Randomized trial of radiotherapy versus concurrent chemoradiotherapy followed by adjuvant chemotherapy in patients with American Joint Committee on Cancer/International Union against cancer stage III and IV nasopharyngeal cancer of the endemic variety. J Clin Oncol.

[5] Lee AW, Lau WH, Tung SY, Chua DT, Chappell R, Xu L, Siu L, Sze WM, Leung TW, Sham JS, Ngan RK, Law SC, Yau TK, Au JS, O'Sullivan B, Pang ES, O SK, Au GK, Lau JT (2005). Preliminary results of a randomized study on therapeutic gain by concurrent chemotherapy for regionally-advanced nasopharyngeal carcinoma: NPC-9901 Trial by the Hong Kong Nasopharyngeal Cancer Study Group. J Clin Oncol.

[6] Baujat B, Audry H, Bourhis J, Chan AT, Onat H, Chua DT, Kwong DL, Al-Sarraf M, Chi KH, Hareyama M, Leung SF, Thephamongkhol K, Pignon JP (2006). Chemotherapy in locally advanced nasopharyngeal carcinoma: an individual patient data meta-analysis of eight randomized trials and 1753 patients. Int J Radiat Oncol Biol Phys.

[7] Wu SG, Liao XL, He ZY, Tang LY, Chen XT, Wang Y, Lin Q (2017). Demographic and clinicopathological characteristics of nasopharyngeal carcinoma and survival outcomes according to age at diagnosis: A population-based analysis. Oral Oncol.

[8] Lee L, Cheung WY, Atkinson E, Krzyzanowska MK (2011). Impact of comorbidity on chemotherapy use and outcomes in solid tumors: a systematic review. J Clin Oncol.

[9] Piccirillo JF, Tierney RM, Costas I, Grove L, Spitznagel EL (2004). Prognostic importance of comorbidity in a hospital-based cancer registry. JAMA.

[10] Birkeland AC, Beesley L, Bellile E, Rosko AJ, Hoesli R, Chinn SB, Shuman AG, Prince ME, Wolf GT, Bradford CR, Brenner JC, Spector ME (2017). Predictors of survival after total laryngectomy for recurrent/persistent laryngeal squamous cell carcinoma. Head Neck.

[11] Asmis TR, Ding K, Seymour L, Shepherd FA, Leighl NB, Winton TL, Whitehead M, Spaans JN, Graham BC, Goss GD (2008). Age and comorbidity as independent prognostic factors in the treatment of non small-cell lung cancer: a review of National Cancer Institute of Canada Clinical Trials Group trials. J Clin Oncol.

[12] Houterman S, Janssen-Heijnen ML, Verheij CD, Louwman WJ, Vreugdenhil G, van der Sangen MJ, Coebergh JW (2004). Comorbidity has negligible impact on treatment and complications but influences survival in breast cancer patients. Br J Cancer.

[13] Kimmick GG, Li X, Fleming ST, Sabatino SA, Wilson JF, Lipscomb J, Cress R, Bergom C, Anderson RT, Wu XC (2018). Risk of cancer death by comorbidity severity and use of adjuvant chemotherapy among women with locoregional breast cancer. J Geriatr Oncol.

[14] Lemmens VE, Janssen-Heijnen ML, Verheij CD, Houterman S, van Driel OJ R, Coebergh JW (2005). Co-morbidity leads to altered treatment and worse survival of elderly patients with colorectal cancer. Br J Surg.

[15] Paleri V, Wight RG (2002). Applicability of the adult comorbidity evaluation - 27 and the Charlson indexes to assess comorbidity by notes extraction in a cohort of United Kingdom patients with head and neck cancer: a retrospective study. J Laryngol Otol.

[16] Sze HC, Ng WT, Chan OS, Shum TC, Chan LL, Lee AW (2012). Radical radiotherapy for nasopharyngeal carcinoma in elderly patients: the importance of co-morbidity assessment. Oral Oncol.

[17] Cao C, Hu Q, Chen X (2018). Intensity-modulated radiotherapy for elderly patients with nasopharyngeal carcinoma. Head Neck.

[18] Yang Q, Zhao TT, Qiang MY, Hu L, Lv X, Ye YF, Ke LR, Yu YH, Qiu WZ, Liu GY, Huang XJ, Li WZ, Lv SH, Sun Y, Zhang LY, Pei F, Guo X, Xiang YQ, Qian CN, Huang BJ, Xia WX (2018). Concurrent Chemoradiotherapy versus Intensity-modulated Radiotherapy Alone for Elderly Nasopharyngeal Carcinoma Patients with Pre-treatment Epstein-Barr Virus DNA: A Cohort Study in an Endemic Area with Long-term Follow-up. J Cancer.

[19] Jin YN, Zhang WJ, Cai XY, Li MS, Lawrence WR, Wang SY, Mai DM, Du YY, Luo DH, Mo HY (2019). The Characteristics and Survival Outcomes in Patients Aged 70 Years and Older with Nasopharyngeal Carcinoma in the Intensity-Modulated Radiotherapy Era. Cancer Res Treat.

[20] Liu H, Chen QY, Guo L, Tang LQ, Mo HY, Zhong ZL, Huang PY, Luo DH, Sun R, Guo X, Cao KJ, Hong MH, Mai HQ (2013). Feasibility and efficacy of chemoradiotherapy for elderly patients with locoregionally advanced nasopharyngeal carcinoma: results from a matched cohort analysis. Radiat Oncol.

[21] Tang LQ, Chen DP, Guo L, Mo HY, Huang Y, Guo SS, Qi B, Tang QN, Wang P, Li XY, Li JB, Liu Q, Gao YH, Xie FY, Liu LT, Li Y, Liu SL, Xie HJ, Liang YJ, Sun XS, Yan JJ, Wu YS, Luo DH, Huang PY, Xiang YQ, Sun R, Chen MY, Lv X, Wang L, Xia WX, Zhao C, Cao KJ, Qian CN, Guo X, Hong MH, Nie ZQ, Chen QY, Mai HQ (2018). Concurrent chemoradiotherapy with nedaplatin versus cisplatin in stage II-IVB nasopharyngeal carcinoma: an open-label, non-inferiority, randomised phase 3 trial. Lancet Oncol.

[22] Lai SZ, Li WF, Chen L, Luo W, Chen YY, Liu LZ, Sun Y, Lin AH, Liu MZ, Ma J (2011). How does intensity-modulated radiotherapy versus conventional two-dimensional radiotherapy influence the treatment results in nasopharyngeal carcinoma patients. Int J Radiat Oncol Biol Phys.

[23] Cox JD, Stetz J, Pajak TF (1995). Toxicity criteria of the Radiation Therapy Oncology Group (RTOG) and the European Organization for Research and Treatment of Cancer (EORTC). Int J Radiat Oncol Biol Phys.

[24] Rubin DB, Thomas N (1996). Matching using estimated propensity scores: relating theory to practice. Biometrics.

[25] Rubin DB (1997). Estimating causal effects from large data sets using propensity scores. Ann Intern Med.

[26] Janssen-Heijnen ML, Houterman S, Lemmens VE, Louwman MW, Maas HA, Coebergh JW (2005). Prognostic impact of increasing age and co-morbidity in cancer patients: a population-based approach. Crit Rev Oncol Hematol.

[27] Wedding U, Röhrig B, Klippstein A, Pientka L, Höffken K (2007). Age, severe comorbidity and functional impairment independently contribute to poor survival in cancer patients. J Cancer Res Clin Oncol.

[28] Ramakrishnan Y, Paleri V, Shah R, Steen IN, Wight RG, Kelly CG (2007). Comorbidity in nasopharyngeal carcinoma: a preliminary communication on the prevalence, descriptive distribution and impact on outcome. Clin Otolaryngol.

[29] Giacalone NJ, Wu J, Chen MH, Renshaw A, Loffredo M, Kantoff PW, D'Amico AV (2016). Prostate-Specific Antigen Failure and Risk of Death Within Comorbidity Subgroups Among Men With Unfavorable-Risk Prostate Cancer Treated in a Randomized Trial. J Clin Oncol.

[30] Lin JC, Liang WM, Jan JS, Jiang RS, Lin AC (2004). Another way to estimate outcome of advanced nasopharyngeal carcinoma-is concurrent chemoradiotherapy adequate. Int J Radiat Oncol Biol Phys.

