# Supplementary material for: The impact of Adult Comorbidity Evaluation-27 on the clinical outcome of elderly nasopharyngeal carcinoma patients treated with chemoradiotherapy or radiotherapy: a matched cohort analysis
Source: J Cancer. 2019 Sep 7;10(23):5614–21. doi: 10.7150/jca.35311 (PMC6843867; doi:10.7150/jca.35311)
Supplement: Supplementary file 1 — Supplementary methods, figure, and table. [file jcav10p5614s1.pdf]

Radiotherapy

All patients were treated with IMRT. The IMRT plan was designed based on previous studies [21, 22] and in accordance with the International Commission on Radiation Units and Measurements, reports 50 and 62. In total, 30-33 fractions were administered at five daily fractions per week for 6-7 weeks. A planning target volume (PTV) was created by adding a three-dimensional margin of 3-5 mm to the delineated target volume to compensate for the uncertainties in treatment set-up and internal organ motion. Gross tumour volume (GTV) included the primary tumour and the enlarged lymph nodes. GTVnx included the sum of the primary tumour volume and the enlarged retropharyngeal nodes, whereas GTVnd was the volume of clinically involved gross cervical lymph nodes. High-risk clinical target volume (CTV1) was defined as the nasopharynx gross tumour volume plus a 5-10 mm margin (2-3 mm posteriorly if adjacent to the brainstem or spinal cord) to encompass the high-risk sites of the microscopic extension and the whole nasopharynx. Low-risk clinical target volume (CTV2) was defined as the high-risk clinical target volume plus a 5-10 mm margin (2-3 mm posteriorly if adjacent to the brainstem or spinal cord) to encompass the low-risk sites of the microscopic extension. These included the base of the skull, clivus, sphenoid sinus, parapharyngeal space, pterygoid fossae, posterior parts of the nasal cavity, pterygopalatine fossae, retropharyngeal nodal regions, and the elective neck area from level IB to level V. The prescribed doses for the PTV derived from GTVnx, GTVnd, CTV1, and CTV2 were 66-70 Gy, 64-70 Gy, 60-62 Gy, and 54-56 Gy, respectively.

Chemotherapy

Patients from the CRT group received cisplatin-based concurrent chemotherapy with or without sequential chemotherapy. Cisplatin was administered at 80 mg/m^2 3-weekly or at 40 mg/m^2 weekly. Sequential chemotherapy regimens included neoadjuvant and/or adjuvant

chemotherapy. The most commonly used regimens for neoadjuvant chemotherapy were cisplatin (80 mg/m^2) with docetaxel (80 mg/m^2) every 3 weeks or cisplatin (80 mg/m^2) with 5-fluorouracil (800 mg/m^2 daily, over a 5-day period). Selection of sequential chemotherapy and concurrent regimens were considered by the individual doctors. If necessary, dose modification was performed at the doctors' discretion. Chemotherapy was not given to some patients for the following conditions: patient refusal, older than 75 years, and intolerance or contraindication to chemotherapy.

Table S1 Clinical characteristics in validation cohort

	Validation cohort (n = 72)		
Characteristics	RT	CRT	P-value
**Total**	36	36	
**Age, y**			
<75	23(63.8%)	25(69.4%)	0.803
≥75	13(36.2%)	11(30.6%)	
**Gender**			
Female	11(30.5%)	9(25.0%)	0.793
Male	25(69.5%)	27(75.0%)	
**T stage#**			
T1-2	9(25.0%)	11(30.6%)	0.793
T3-4	27(75.0%)	25(69.4%)	
**N stage#**			
N0-1	10(27.8%)	7(19.4%)	0.580
N2-3	26(72.2%)	29(80.6%)	
**TNM stage#**			
III	24(66.7%)	19(52.8%)	0.337

IV	12(33.3%)	17(47.2%)	
**RT dose (NP)**			
<70 Gy	1(2.8%)	2(5.6%)	0.572*
≥70 Gy	35(97.2%)	34(94.4%)	
**RT dose (LN)**			
<60 Gy	13(33.0%)	11(27.5%)	0.803
≥60 Gy	23(67.0%)	25(72.5%)	

Abbreviations: CRT = chemoradiotherpy; RT = radiotherapy ; NP = nasopharynx; LN = lymph node

According to the 7th edition of UICC/AJCC staging system

P-value was calculated with the Pearson $\chi 2$ test or Fisher's exact test (*)

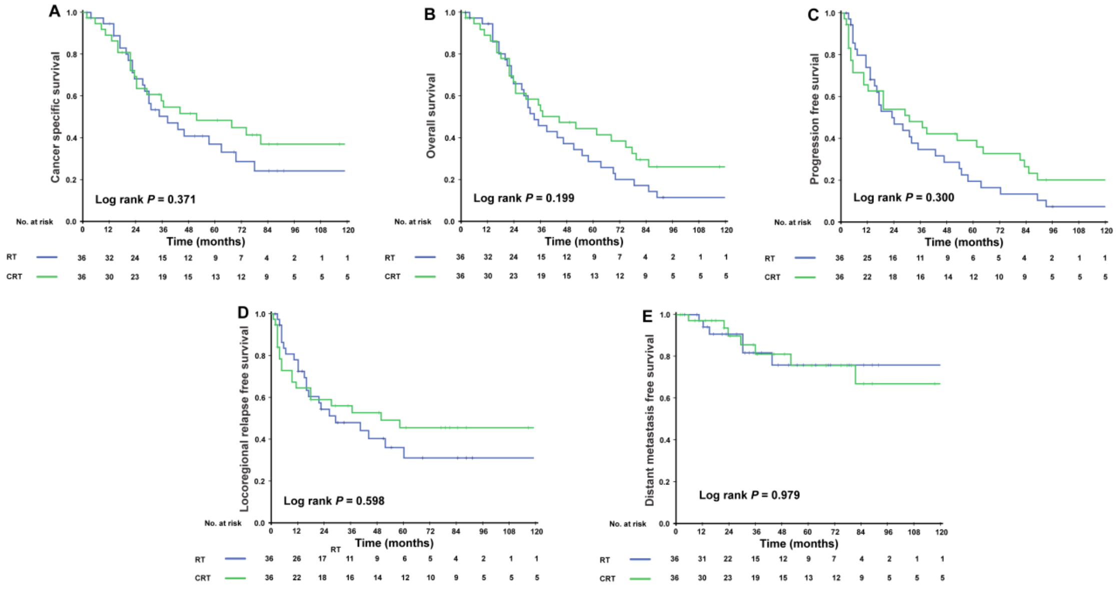

Figure S1: Kaplan–Meier survival curves between the RT and CRT groups in elderly patients in the validation cohort. Shown are results for (A) cancer-specific survival, (B) overall survival, (C) progression-free survival, (D) locoregional relapse-free survival, (E) distant metastasis free survival. P values were calculated using the unadjusted log-rank test.
